# Isolation of Cell Nuclei Using Inert Macromolecules to Mimic the Crowded Cytoplasm

**DOI:** 10.1371/journal.pone.0007560

**Published:** 2009-10-23

**Authors:** Ronald Hancock, Yasmina Hadj-Sahraoui

**Affiliations:** Laval University Cancer Research Centre, Hôtel-Dieu Hospital, Québec, Québec, Canada; Institut Pasteur Korea, Republic of Korea

## Abstract

Cell nuclei are commonly isolated and studied in media which include millimolar concentrations of cations, which conserve the nuclear volume by screening the negative charges on chromatin and maintaining its compaction. However, two factors question if these ionic conditions correctly reproduce the environment of nuclei in vivo: the small-scale motion and conformation of chromatin in vivo are not reproduced in isolated nuclei, and experiments and theory suggest that small ions in the cytoplasm are not free in the soluble phase but are predominantly bound to macromolecules. We studied the possible role in maintaining the structure and functions of nuclei in vivo of a further but frequently overlooked property of the cytoplasm, the crowding or osmotic effects caused by diffusible macromolecules whose concentration, measured in several studies, is in the range of 130 mg/ml. Nuclei which conserved their volume in the cell and their ultrastructure seen by electron microscopy were released from K562 cells in media containing the inert polymer 70 kDa Ficoll (50% w/v) or 70 kDa dextran (35% w/v) to replace the diffusible cytoplasmic molecules which were dispersed on cell lysis with digitonin, with 100 µM K-Hepes buffer as the only source of ions. Immunofluorescence labelling and experiments using cells expressing GFP-fusion proteins showed that internal compartments (nucleoli, PML and coiled bodies, foci of RNA polymerase II) were conserved in these nuclei, and nascent RNA transcripts could be elongated. Our observations are consistent with the hypothesis that crowding by diffusible cytoplasmic macromolecules is a crucial but overlooked factor which supports the nucleus in vivo by equilibrating the opposing osmotic pressure cause by the high concentration of macromolecules in the nucleus, and suggest that crowded media provide more physiological conditions to study nuclear structure and functions. They may also help to resolve the long-standing paradox that the small-scale motion and irregular conformation of chromatin seen in vivo are not reproduced in nuclei isolated in conventional ionic media.

## Introduction

Cell nuclei are commonly isolated and studied in media which have evolved from empirical observations of the conditions which maintain their ultrastructural features and functions such as transcription [1]–[6], or are based on the estimated concentrations of ions in the cell [5]. In these media, the volume of nuclei is conserved by including cations which screen the negative charges on chromatin and thus maintain its compaction [7], [8]; without these cations, nuclei swell and may lyse [1], [6], [8], [9] due to the osmotic pressure exerted by their high internal concentration of macromolecules [10].

It is tacitly believed that the stability and functioning of nuclei in these media reflects the fact that they reproduce the ionic environment in the cytoplasm which surrounds the nucleus in vivo, but this idea is not consistent with all the available experimental evidence. Firstly, persuasive biophysical considerations suggest that the cytoplasm in vivo contains no *free* ions ([11]. Both K^+^ [12]–[14] and Na^+^ ions [12], [15] in the cell are believed to be bound predominantly to macromolecules, and Mg^2+^ ions to ATP and probably also to negatively-charged macromolecules [11] or are sequestered by mitochondria and the sarcoplasmic reticulum [16]–[18]. Secondly, chromatin isolated from nuclei prepared in conventional ionic media, or reconstituted in similar conditions, does not reproduce completely the properties of chromatin within the cell which shows constrained motion on a 100 nm-scale [19] and an irregular conformation [20]–[22], whereas in isolated nuclei the small-scale motion of chromatin is supressed [19] and chromatin isolated from them has a regular and symmetrical conformation termed the 30 nm fibre [23]. The binding of significant amounts of Mg^2+^ and Ca^2+^ ions to nuclei, and possibly to chromatin, during their isolation in ionic media [24] may contribute to this discrepancy.

These data suggest that the volume and structure of the nucleus in the cell are maintained by other factors, and not by cations at the mM concentrations which are used in conventional nuclear isolation media. Here we have considered the importance of the property of the cytoplasm which is frequently termed its “crowded” nature [25]–[34]. The concentration of diffusible macromolecules in the fluid phase of the cytoplasm is in the range of 130 mg/ml [26]–[29]; this is of course a global value averaged over many local microenvironments with, for example, different spatial and temporal extents of polymerisation of macromolecules which form cytoskeletal elements [27], [33], but nevertheless at this concentration of diffusible macromolecules organelles and macromolecular assemblies in the cytoplasm are predicted to experience quite strong crowding effects [35]–[37]. More information about the size distribution of diffusible macromolecules is needed for precise computation of these effects, but direct measurement shows that the osmotic pressure in frog muscle cells is equivalent to ∼170 mg/ml of bovine serum albumin [27]. That crowding effects are indeed important in the cytoplasm has been recognised in other contexts, for example to explain the anomalous diffusion of tracer macromolecules [30], [38] and the monomer-polymer equilibria in vivo of actin [39] and of other cytoplasmic filament proteins [40]. Proteins or inert polymers which mimic crowding effets in the cytoplasm are commonly included in isolation media to maintain the structure and functions of mitochondria [41]–[43] and peroxisomes [44].

Macromolecular crowding effects can be understood alternatively in terms of osmotic forces [45], [46], which in the case of nuclei represent an osmotic pressure exerted by cytoplasmic macromolecules on the exterior of the nuclear envelope, which is semipermeable and excludes macromolecules larger than ∼70 kDa except those selected to enter through nuclear pores [47],[48]. Here we show that nuclei which conserve their structure, internal compartmentalisation, and transcriptional activity can be isolated in media which contain an inert, volume-occupying polymer to replace the cytoplasmic macromolecules which are dispersed upon cell breakage, with no ionic components other than 100 µM K-Hepes buffer. These observations are consistent with the idea that crowding forces due to cytoplasmic macromolecules are an important but overlooked parameter which supports the structure and functions of the nucleus in vivo.

## Results

### Isolation and structure of nuclei in media containing an inert polymer

For isolating nuclei we used media which contained uncharged, inert polymers which have been used widely to study effects of crowding on macromolecular systems [37], [49]–[53]. The polymers were Ficoll (average M_r_ 70 kDa), an approximately spherical branched and crosslinked polymer of sucrose [54], and dextran (average M_r_ 69.9 kDa), a flexible linear random coil polymer of glucose [55]. Macromolecules of this size cannot traverse the nuclear envelope [47], [48], [56] and therefore exert a crowding or osmotic effect on the exterior of nuclei. The only ionic component of the polymer solutions was 100 µM K-Hepes buffer, pH 7.4. To maintain the volume of isolated nuclei at the level in vivo, polymer solutions which were close to saturation were required and at their density and viscosity cells could not be washed by centrifugation; therefore, after pelleting growing cells the medium was carefully removed and the cells were resuspended directly in polymer solution supplemented with digitonin, which permeabilises the cytoplasmic membrane while conserving the nuclear membrane [47]. After mixing on a vortexer at maximum speed to disperse cytoplasmic material, nuclei were released and were centrifuged onto slides for optical microscopy or pelleted for electron microscopy.

**Figure 1:**
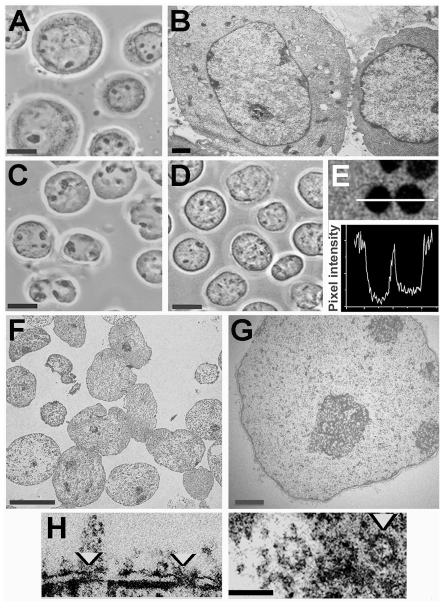
Nuclei of K562 cells released in polymer solution containing 100 µM K-Hepes. Cells suspended in 70 kDa Ficoll (50% w/v) or 70 kDa dextran (35% w/v) containing digitonin were vortexed to disperse cytoplasmic material, and nuclei were centrifuged and fixed in the same polymer solution. (A) intact cells in growth medium, phase contrast. (B) intact cells, electron microscope section. (C, D) nuclei isolated in Ficoll or dextran respectively, phase contrast. (E) nuclei incubated as in D in dextran with addition of fluorescein-labelled dextran of the same size, fluorescence image of an approximately midplane 0.5 µm confocal section after 1 h; lower panel shows a scan of fluorescence intensity along the white line. (F, G) nuclei isolated in Ficoll and processed as in B, electron microscope images of sections. (H) nuclear pores (arrowheads) in nuclei isolated in Ficoll seen in transverse (left) and tangential (right) electron microscope sections. Bars 10 µm (A, C, D, F); 1 µm (B, G); 0.2 µm (H).

Nuclei which were structurally well conserved and free of cytoplasmic material as seen by phase-contrast and electron microscopy were released in solution containing 50% Ficoll or 35% dextran and 100 µM K-Hepes buffer (Figure 1). In these nuclei the nucleoli, which are prominent in phase-contrast images of intact K562 cells (Figure 1A), were conserved (Figure 1C, D) and were seen as more electron-dense structures by electron microscopy (Figure 1F, G). The rather homogeneous distribution of chromatin in isolated nuclei (Figure 1F, G) resembled that in the nuclei of intact cells processed in the same conditions (Figure 1B). The main features of the nuclear envelope and pores were conserved in isolated nuclei (Figure 1H). Nuclei of comparable purity and ultrastructure were released from Raji cells in the same conditions (unpublished results). We confirmed that polymers of this size are excluded from nuclei in the conditions used here, as observed in other studies [47], [56]: nuclei isolated in dextran and deposited on slides were incubated in the same polymer solution containing fluorescein-labelled dextran of the same size, and linescans of the fluorescence signal were recorded across approximately mid-plane confocal sections of nuclei (Figure 1E). The mean fluorescence intensity in the nuclear interior was 3.2±0.4% (SEM, n = 10) of that in the external medium after incubation for 30 min, and 12±6% after 1 h.

### Nuclear volume responds to the concentration of polymer

**Figure 2:**
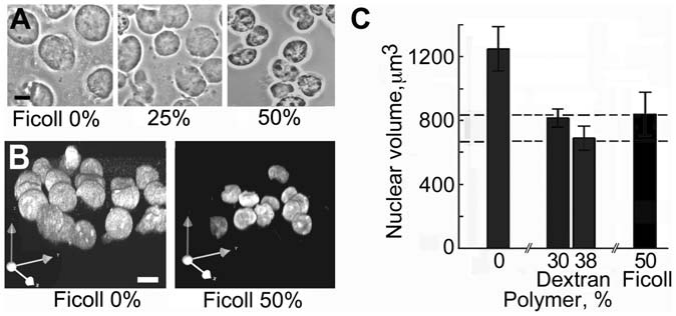
Influence of the concentration of polymer in the medium on nuclear volume. Nuclei were released as described in Figure 1, centrifuged onto slides, incubated for 30 min in a solution of the same polymer at the concentration shown, and fixed in the same solution. (A) nuclei in Ficoll, phase-contrast. (B) nuclei in Ficoll stained with YOYO, 3D volumes rendered from serial confocal sections to confirm that the changes of diameter in (A) represent changes of volume. (C) mean nuclear volume as a function of polymer concentration. Error bars show 95% confidence limits (n = 30) and horizontal dashed lines 95% confidence limits for the volume of nuclei in living cells labelled with DRAQ5. All differences of mean volume from that in 0% polymer were significant at p≤0.01, calculated by the Welch t-test. Bars 10 µm.

The mean volume of nuclei in 50% Ficoll or 35% dextran was 840±220 µm^3^ or 680±210 µm^3^, respectively (SEM, n = 30), compared with their volume of 750±210 µm^3^ (SEM, n = 30) measured in living cells. The nuclear volume responded to the concentration of polymer in the medium (Figure 2), and these changes of volume were reversible (unpublished results).

### The internal compartmentalisation of nuclei is conserved in polymer solutions

**Figure 3:**
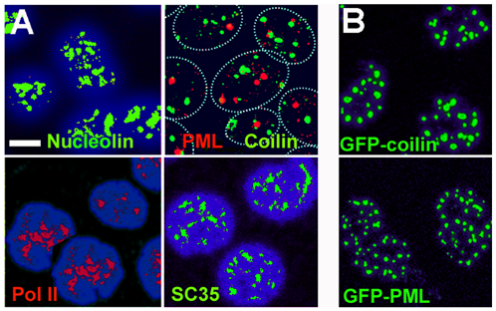
Internal compartments in nuclei isolated in 50% Ficoll, 100 µM K-Hepes. (A) distribution of nucleolin (nucleoli), PML (PML bodies), coilin (Cajal bodies), RNA polymerase II (pol II) (transcription factories) and SC35 (speckles). Nuclei of K562 cells were isolated in polymer solution, deposited on slides, and fixed as described in Figure 1. In the upper right panel, approximate nuclear outlines were traced from an overexposed image. (B) distribution of GFP-coilin or GFP-PML isoform IV fusion proteins in nuclei of HeLa or U2OS cells, respectively. Cells were washed with polymer solution and permeabilised and extracted in situ. Images are maximum intensity projections from deconvoluted confocal series. Bar 5 µm.

Within the nucleus, many macromolecules form regions of high local concentration which are termed compartments and include nucleoli, PML and Cajal bodies, speckles, transcription factories, and other types [57], [58]. As an indication of the global conservation of nuclear architecture in nuclei isolated in polymer solution, we examined these compartments by immunofluorescence labelling of their characteristic protein components. In nuclei isolated in 50% Ficoll (Figure 3) and in 35% dextran (unpublished results), all the compartments examined (nucleoli, PML and Cajal bodies, speckles, and transcription factories) were conserved. Mean numbers of 4.9 nucleoli, 4.7 PML bodies, and 1.5 Cajal bodies were counted in nuclei of cells isolated in 50% Ficoll (n = 100), whereas the corresponding numbers were 4.2, 4.5, and 1.4 respectively in nuclei isolated from the same cells in a conventional ionic medium [5].

Experiments with cells expressing a GFP-fusion of the protein PML isoform IV or of coilin (Figure 3B) confirmed that PML and Cajal bodies were conserved in nuclei isolated in polymer solution; these cells were washed with polymer solution and permeabilised and extracted in situ because after detachment by trypsin, digitonin lysis, and deposition on slides their nuclei were masked by cosedimenting extracellular matrix material. The mean number of PML bodies in nuclei isolated from cells expressing GFP-PML isoform IV was ∼20 (n = 100), close to the value of ∼22 reported in living cells [59], while in nuclei isolated from cells expressing GFP-coilin the mean number of Cajal bodies was ∼12, compared with 4–5 observed in living cells by Platini et al [60].

**Figure 4:**
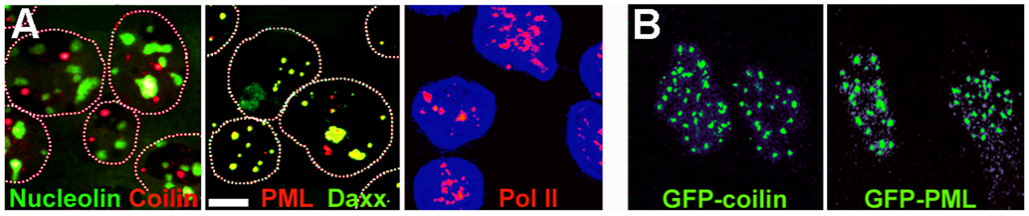
Compartments in nuclei isolated in 50% Ficoll, 100 µM K-Hepes by cell lysis with 0.5% Triton X-100 to permeabilise the nuclear membrane. (A) proteins characteristic of different compartments visualised by immunofluorescence as described in Figure 3. Daxx is a further component of PML bodies. (B) GFP-coilin or GFP-PML isoform IV fusion proteins in nuclei of HeLa or U2OS cells, respectively. In the two left panels, approximate nuclear outlines were traced from overexposed images. Images are maximum intensity projections from deconvoluted confocal series. Bar 5 µm.

The conservation of Cajal bodies and nucleoli in nuclei in polymer solutions which contained only 100 µM K-Hepes buffer (Figure 3) was unexpected, because after their isolation Cajal bodies require Mg^2+^ at 0.5 mM for their stability [61] and nucleoli require Mg^2+^, Ca^2+^ and/or polyamines at concentrations of 0.2–3 mM [62]–[64]. This discrepancy could be understood if these compartments were stabilised by free cations or polyamines in pools which were conserved within nuclei isolated using digitonin. We therefore examined the effect of replacing digitonin for cell lysis by Triton X-100, an ionic surfactant which removes the membrane components of the nuclear envelope [65] and therefore should allow any free ions to escape. Nucleoli, PML and Cajal bodies, and RNA pol II foci were still present in nuclei isolated by Triton lysis in 50% Ficoll (Figure 4) and in 35% dextran (unpublished results).

### Nuclei isolated in polymer solution conserve transcriptional activity

**Figure 5:**
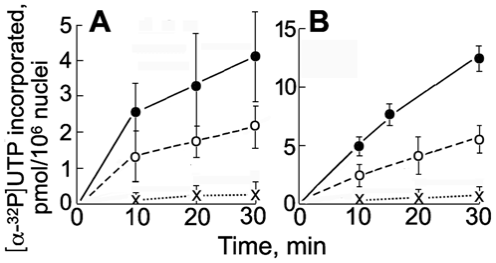
Run-on incorporation of [α-^32^P]UTP into nascent RNA in nuclei isolated and incubated in polymer solution in 100 µM K-Hepes. K^+^ was added to the medium to ∼1 mM to neutralise the nucleotide triphosphate RNA precursors. (A) nuclei isolated and incubated in 50% Ficoll; data points and error bars show means ±SEM (n = 5) for 30 min, and means and ranges (n = 2) for other points. (B) nuclei isolated and incubated in 12% 8 kDa PEG; data points and error bars show means ±SEM (n = 9 for 30 min, n = 3 for other points). Full lines show incorporation in the complete system, dashed lines with addition of α-amanitin (200 µg/ml) to inhibit RNA pol II, and dotted lines in the absence of polymer.

In nuclei isolated in polymer solution, RNA pol II was still concentrated in foci (Figure 3) which could represent transcription factories where RNA pol II-mediated transcription occurs [58], and we tested if these nuclei could incorporate [α-^32^P]UTP into elongating RNA chains. For these experiments, the K^+^ concentration in the transcription medium had to be increased to ∼1 mM to neutralise the nucleotide triphosphate RNA precursors. Nuclei isolated and incubated in 50% Ficoll incorporated [α-^32^P]UTP at an initial rate of ∼0.25 pmoles UTP/min/10^6^ nuclei (Figure 5A), and a similar rate was seen in nuclei isolated in 35% dextran (unpublished results). For comparison, the rate of incoporation was ∼0.5 pmoles UTP/min/10^6^ in nuclei isolated and incubated in an ionic medium [5] with the same RNA precursors (unpublished results) and ∼1 pmole/min/10^6^ cells after permeabilisation by Triton X-100 [66], [67]. Run-on transcription was essentially polymer-dependent; in the absence of polymer the rate was <5% of that when a polymer was present (Figure 5). Inhibition of RNA pol II by α-amanitin reduced the incorporation at 30 min to 63±26% (SEM, n = 5) of the control value (Figure 5A).

### Isolation of nuclei in medium containing a polymer which permeates the nuclear envelope

**Figure 6:**
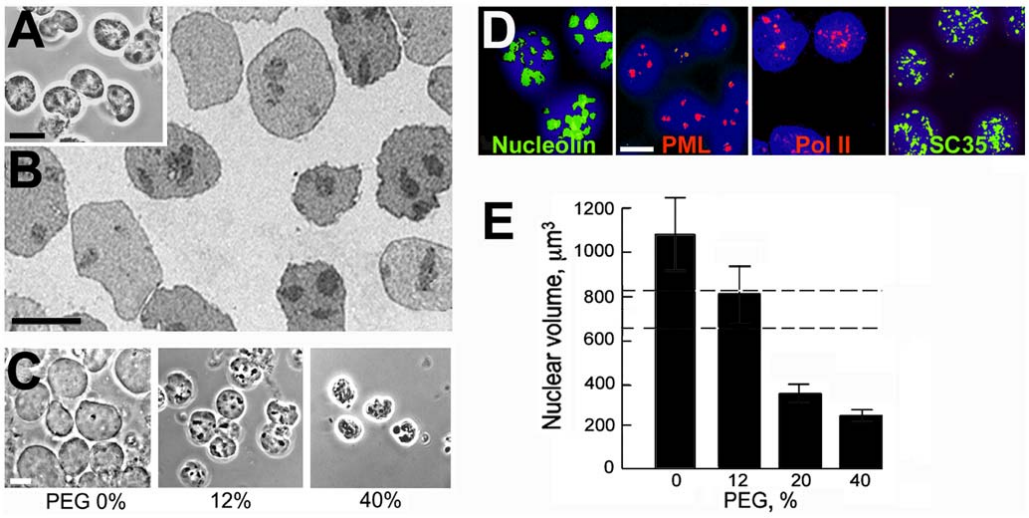
Nuclei isolated in solution containing the permeant polymer 8 kDa PEG and 100 µM K-Hepes. Cells were washed in 12% PEG, and nuclei were released by homogenisation in the the same solution containing digitonin and centrifuged onto slides or processed for electron microscopy. (A) nuclei in 12% PEG, phase-contrast. (B) sectioned nuclei in 12% PEG, electron microscopy. (C) nuclei deposited on slides and incubated for 30 min with PEG at the concentration shown in 100 µM K-Hepes, pH 7.4, phase-contrast. (D) proteins characteristic of compartments in nuclei isolated and incubated in 12% PEG, visualised by immunofluorescence as described in Figure 3. Images are maximum intensity projections from deconvoluted confocal series. (E) mean nuclear volume (n = 30) as a function of concentration of PEG; error bars are as defined in Figure 2, and dashed lines show 95% confidence limits for the volume of nuclei in living cells. All differences of nuclear volume from that in 0% PEG were significant at p≤0.005. Bars 10 µm.

Handling and pipetting Ficoll and dextran solutions at concentrations which maintained the volume of isolated nuclei was inconvenient due to their viscosity and density, and we therefore explored if these could be replaced by a less viscous polymer; this implied, of course, that the polymer should be of smaller size and consequently would permeate through the nuclear envelope and exert crowding effects on macromolecules within the nucleus. Nuclei could be isolated using 12% 8 kDa poly(ethylene glycol) (PEG) in 100 µM K-Hepes, pH 7.4; their appearance in phase-contrast and electron microscope images was similar to that of nuclei isolated in a non-permeating polymer (Figure 6A, B) and the internal compartments examined were conserved (Figure 6D). Their volume was 760±180 µm^3^ (SEM, n = 60) compared with 750±210 µm^3^ (SEM, n = 30) in living cells.

Nascent RNA transcripts were elongated in nuclei isolated in 12% PEG at an initial rate ∼two- fold greater than that in nuclei isolated in 50% Ficoll (Figure 5B). The nuclear volume varied over an ∼four-fold range with the concentration of PEG (Figure 6E), offering an interesting experimental approach to manipulate nuclear volume; at the highest concentration of PEG tested (40%) nuclei contracted and their periphery became crenellated (Figure 6C). Similar properties were seen in nuclei isolated in 10.5 kDa dextran (12%) (unpublished results).

## Discussion

In this work, we show that cell nuclei which conserve their ultrastructure, internal compartmentalisation, and transcriptional activity can be stabilised by osmotic effects in solutions where an inert, volume-occupying polymer at an appropriate concentration replaces the diffusible cytoplasmic macromolecules which are dispersed upon cell lysis, and that they do not require the mM concentrations of cations which are commonly used in isolation media. The conservation of the structure of nuclei in these conditions is entirely consistent with the well-documented effects of macromolecular crowding in favouring the formation and stability of assemblies of macromolecules [35]–[37], [49]–[53]. Further, the ability of a crowded environment to replace ions for conserving the structure and functions of nuclei is quite typical of a growing number of observations on macromolecular assemblies, although the nucleus is probably the largest assembly for which this phenomenon has been observed. Crowding agents can replace the requirement for a high ionic strength to polymerise the capsid protein CA of HIV-1 [53] and attenuate the need for Mg^2+^ ions for the association between 30S and 50S ribosomal subunits [49] and of K^+^ ions for productive folding of the chaperonin GroEL [51], and isolated chromosomes of *Escherichia coli* [50], [52] and of eukaryotic cells (unpublished results) are stabilised by inert polymers which reproduce their crowded environment in vivo, without the divalent cations and/or polyamines which were used earlier. The dependence of transcription on the addition of an inert polymer in nuclei isolated in 100 µM K-Hepes observed here is consistent with a crowding-mediated assembly and functioning of the transcriptional machinery, a model which was first proposed to understand the requirement for polyvinylpyrrolidone or Ficoll to prepare nuclear extracts which initiate RNA pol II transcription correctly [68]; transcription in permeabilised cells in an ionic buffer is also stimulated by PEG and BSA [69] or Ficoll [70], although the underlying mechanism was not discussed.

Does the conservation of the structure and functions of the nucleus by crowding or osmotic forces observed here play a significant role in the cell? A considerable body of experimental evidence supports this hypothesis. Firstly, crowding effects do indeed occur in the cytoplasm in vivo, as deduced from the anomalous diffusion of tracer macromolecules [30], [38], and the volume of the nucleus in vivo is modulated by the concentration of macromolecules in the cytoplasm; nuclei of amphibian oocytes contract when BSA or polyvinylpyrrolidone are injected into the cytoplasm [71] and the effects of solute molecules on nuclear size in permeabilised macrophages led to the conclusion that “high concentrations of macromolecules such as those found inside cells can influence the size of the nucleus by directly affecting nuclear structure” [72]. Secondly, consideration of quantitative data shows that the polymer solutions which were found here to maintain the volume of isolated nuclei close to that in vivo reproduce quite closely the osmotic pressure which diffusible cytoplasmic macromolecules are predicted to exert on the nucleus in vivo; 50% 70 kDa Ficoll and 35% 70 kDa dextran solutions have an osmotic pressure of ∼210 and ∼270 kPa, respectively [73], [74] whereas a value of ∼200 kPa was measured directly in the cytoplasm of frog muscle [27]. This latter value is equivalent to that of a solution of BSA at ∼170 mg/ml [75], whereas the concentration of diffusible macromolecules is ∼130 mg/ml in the fluid phase of the cytoplasm of rabbit muscle cells [26] and that of the fifteen most abundant diffusible proteins alone is 82 mg/ml in muscle fibres [29]. These high values are not unique to muscle cells, and in fibroblasts the diffusion of Ficoll in the cytoplasm resembles that in a solution containing 124 mg/ml of “background” macromolecules in the fluid phase together with 37 mg/ml of filamentous macromolecules [28].

Thirdly, these arguments have to be considered in the light of the doubts, cited in the Introduction, if media currently used to conserve the stability and functions of nuclei reproduce the ionic properties of the cytoplasm in vivo. Levels of cytoplasmic ions measured by biophysical methods cannot be interpreted as concentrations without knowing the extent to which they are bound to charged macromolecules, which itself can cause uncertainty in the calibration of analytical methods [12], [16], [18]. Indeed, a strong case has been made that the cytoplasm does not contain *free* small ions at significant levels and that “there is no cytoplasmic ‘bulk’ concentration of ions and metabolites (as is often assumed in biophysical models and in the design and interpretation of in vitro experiments)” [11]. However, nuclei isolated in conventional ionic media avidly bind extra Mg^2+^ and Ca^2+^ from buffers to levels many-fold the endogenous levels [24], an effect which could provide clues to resolving the paradox cited in the Introduction that chromatin from nuclei isolated in ionic media, or reconstituted in similar conditions, does not reproduce completely the properties of chromatin within the nucleus in vivo.

That nuclei can be stabilised by osmotic effects has been observed, in fact, since sucrose was first used in isolation media [76] and inert macromolecules or BSA have been used previously in media for isolating nuclei [77]–[81]. However, in general Arthur Kornberg's 7th commandment “Correct for extract dilution with molecular crowding” [82] has been overlooked when isolating and studying nuclei and their components. The specific features of the macromolecular interactions which occur in the nucleus in vivo may not be completely reproduced by inert crowding agents, but we propose that their use in media to isolate and handle nuclei and their components should provide an environment for studies of structures and processes which corresponds more closely to that in the cell.

## Materials and Methods

### Cells

K562 (human erythroleukemia) cells were grown in suspension culture in DMEM, U2OS cells stably expressing GFP-PML isoform IV as monolayers in DMEM with G418 (1600 µg/ml; Invitrogen), and HeLa cells inducibly expressing GFP-coilin [60] as monolayers with transfer to tetracycline-free medium for 4–6 h to allow GFP-coilin expression. Media contained 10% FCS (Invitrogen), 100 units/ml penicillin, and 100 µg/ml streptomycin. Cells were harvested when cultures reached ∼half the maximum cell density.

### Polymer solutions

Ficoll (average M_r_ 70 kDa, Fluka), dextran (average M_r_ 69.9 kDa, Sigma-Aldrich), and PEG (average M_r_ 8 kDa, Fluka molecular biology grade) were dissolved in bidistilled H_2_O at 60–70°C and the solutions were deionised by shaking with the mixed-bed resin AG 501-X8(D) (Bio-Rad) for 6–8 h. K-Hepes (Sigma-Aldrich) buffer, pH 7.4 was added to a final concentration of 100 µM and solutions were passed through an 0.45 µm filter and stored at −20°C; their pH was verified before each experiment. Polymer M_r_ values are from the suppliers and concentrations are expressed as w/v.

### Isolation of nuclei

All steps were at room temperature (∼20°C) and cells and nuclei were handled using cut-off micropipette tips. Using 50% Ficoll or 35% dextran solutions, cells could not be washed by centrifugation because of their high density and viscosity and they were centrifuged (60 *g*, 6 min), the growth medium was removed carefully by micropipette and then with an absorbent paper wick, and the cells were resuspended at ∼1–5×10^6^ cells/ml in polymer solution supplemented with 100 µg/ml digitonin (Boehringer, high purity) diluted from a stock solution of 10 mg/ml in H_2_O prepared fresh every month. Using PEG solution, cells were washed in 12% PEG by centrifugation (600 *g*, 10 min) and resuspended in PEG solution supplemented with digitonin. In one series of experiments, digitonin was replaced by 0.5% v/v Triton X-100 (Sigma-Aldrich). After 10 min, cells in Ficoll or dextran were mixed on a vortexer at maximum speed for 2 to 3 min; cells in PEG could be broken in the same way or by ∼50 gentle hand strokes in a 2 ml glass homogeniser with a teflon piston (Wheaton), until >95% of the cells had released their nucleus as seen by phase-contrast microscopy. Two volumes of the same polymer solution without digitonin were added, and aliquots of the suspension containing ∼10^6^ nuclei were centrifuged onto polylysine-coated slides in a cytological centrifuge (Cytospin 2, Shandon) (4000 *g*, 40 min in Ficoll or dextran or 1000 *g*, 10 min in PEG). These methods could not be used for adherent HeLa and U2OS cells because after detachment by trypsin, digitonin lysis, and deposition on slides their nuclei were masked by cosedimenting extracellular matrix material; instead, they were grown on cover glasses, growth medium was removed carefully with absorbent paper, and the cells were washed with polymer solution and then permeabilised and extracted in situ in 500 µl of the same solution containing 50 µg/ml digitonin or 0.5% v/v Triton X-100 for 30 min. Nuclei were incubated in polymer solutions of different concentrations after depositing them on slides; they were overlayed with 200 µl of polymer solution and incubated in a humidified box for 30 min at room temperature. To examine the exclusion of dextran from nuclei, nuclei deposited on a slide were incubated for 30 min or 1 h in 35% dextran solution in which 10% of the dextran was replaced by fluorescein-labelled 70 kDa dextran (Sigma-Aldrich).

### Run-on transcription

Nuclei isolated in polymer solution from ∼10^6^ cells were centrifuged in 15 ml conical polypropylene tubes (VWR) (5000 *g*, 15 min in Ficoll or dextran or 2200 *g*, 5 min in PEG), resuspended in 50 µl of the same polymer solution, and equilibrated at 35°C for 10 min. Five µl of a transcription mix prepared in the same polymer solution were added to give final concentrations of 100 µM ATP, CTP, GTP, and UTP, 10 units/ml RNAguard (GE Healthcare), and 20 µCi [α-^32^P]UTP (6000 Ci/mmol; Perkin-Elmer), and samples were mixed and incubated at 35°C. To inhibit RNA pol II, α-amanitin (200 µg/ml; Sigma-Aldrich) was added to the suspension of nuclei 10 min before adding the transcription mix. Incorporation was arrested by adding 10% TCA to duplicate samples, and after 30 min on ice the precipitates were collected on GF/B filters, washed exhaustively with 10% TCA containing disodium pyrophosphate (10 g/l) followed by 70% ethanol, dried, and subjected to liquid scintillation counting.

### Immunofluorescence

Nuclei deposited on slides were fixed by overlaying them for 10 min with the same polymer solution as the preceding step containing 2% paraformaldehyde, permeabilised with 0.5% Triton X-100 in PBS for 10 min, and incubated overnight at 4°C in casein blocking reagent (Roche). All subsequent incubations were at room temperature in a humidified box, and washes and antibody dilutions were in PBS. Slides were incubated for 1 h with primary antibodies recognising nucleolin (Research Diagnostics, mAb; dilution 1/100 or 1/200), RNA pol II (M. Vincent, mAb CC-3; 1/100), SC35 (Sigma-Aldrich, mAb S-4045; 1/100), PML (Santa Cruz, mAb sc-966; 1/1000); coilin (E.K.L. Chan, rabbit R288; 1/100), or Daxx (Santa Cruz, rabbit sc-7152; 1/100). After washing, slides were incubated for 1 h with an appropriate secondary antibody conjugated with Alexa 488, 568, or 594 (Molecular Probes) diluted 1/100, and washed again. GFP fusion proteins were visualised after fixing nuclei as described above. DNA was stained with DAPI and preparations were mounted in SlowFade (Molecular Probes).

### Imaging

Serial 0.5 µm or 2 µm confocal sections were acquired on an MRC1024 (BioRad) with a 60x NA 1.4 oil-immersion objective or an Ultraview ERS (Perkin-Elmer) with a 60x NA 1.4 Plan Apo objective, and phase-contrast images on a Nikon E800 with a 100x NA 1.3 oil-immersion objective and a CoolSNAP camera (Roper Scientific). To measure nuclear volumes, living cells were labelled for 10 min with 1 µM DRAQ5 (Biostatus, UK) and fixed cells with 10 nM YOYO-1 (Molecular Probes) or 0.5 µg/ml propidium iodide in PBS. Stacks of 0.5 µm confocal sections were acquired from 30 randomly-selected nuclei and their areas in successive sections were summed. Differences between mean nuclear volumes were assessed by the Welch t-test to calculate 95% confidence intervals and p-values. Deconvolution, maximum intensity projections, area measurements, and linescans across 0.5 µm confocal sections through nuclei incubated with fluorescein-labelled dextran were made using Metamorph (Universal Imaging), 3D volumes were rendered using Volocity 4 (Improvision), and Photoshop 7.0 was used to pseudocolour and merge images and for assembly of Figures. When shown, approximate nuclear outlines were traced from an overexposed image. For examination by electron microscopy, nuclei isolated in polymer solution were pelleted (5000 *g*, 30 min in Ficoll and dextran or 700 *g*, 10 min in PEG) and suspended in the same polymer solution; nuclei isolated in “physiological buffer” [5] were pelleted (200 *g*,10 min) and resuspended in the same buffer. After fixation by addition of paraformaldehyde (2%) and glutaraldehyde (0.1%) for 1 h on ice, the nuclei were centrifuged and embedded in 1.5% low melting point agarose (Sigma-Aldrich) in PBS. Fragments were cut from the agarose, dehydrated, embedded in Poly/Bed 812 (Polysciences), and 90 to100 nm-thick sections were placed on nickel or copper grids and stained with uranyl acetate and lead citrate. Digital images were acquired on a Jeol 1200 electron microscope.
